# ^18^F-FDG PET as novel imaging biomarker for disease progression after ablation therapy in colorectal liver metastases

**DOI:** 10.1007/s00259-017-3637-0

**Published:** 2017-02-08

**Authors:** M. Samim, W. Prevoo, B. J. de Wit-van der Veen, K. F. Kuhlmann, T. Ruers, R. van Hillegersberg, M. A. A. J. van den Bosch, H. M. Verkooijen, M. G. E. H. Lam, M. P. M. Stokkel

**Affiliations:** 1 Department Surgery, University Medical Centre Utrecht, Heidelberglaan 100, 3508 GA Utrecht, The Netherlands; 2 Department Radiology and Nuclear Medicine, University Medical Centre Utrecht, Heidelberglaan 100, 3508 GA Utrecht, The Netherlands; 3 Department of Radiology and Nuclear Medicine, Antoni van Leeuwenhoek Hospital, Plesmanlaan 121, 1006 BE Amsterdam, The Netherlands; 4 Department Surgical Oncology, Antoni van Leeuwenhoek Hospital, Plesmanlaan 121, 1006 BE Amsterdam, The Netherlands

**Keywords:** RFA, CLM, PERCIST, Imaging biomarker

## Abstract

**Purpose:**

Recurrent disease following thermal ablation therapy is a frequently reported problem. Preoperative identification of patients with high risk of recurrent disease might enable individualized treatment based on patients’ risk profile. The aim of the present work was to investigate the role of metabolic parameters derived from the pre-ablation ^18^F-FDG PET/CT as imaging biomarkers for recurrent disease in patients with colorectal liver metastases (CLM).

**Methods:**

Included in this retrospective study were all consecutive patients with CLM treated with percutaneous or open thermal ablation therapy who had a pre-treatment baseline ^18^F-FDG PET/CT available. Multivariable cox regression for survival analysis was performed using different models for the metabolic parameters (SUL_peak_, SUL_mean_, SUL_max_, partial volume corrected SUL_mean_ (cSUL_mean_), and total lesion glycolysis (TLG)) corrected for tumour and procedure characteristics. The study endpoints were defined as local tumour progression free survival (LTP-FS), new intrahepatic recurrence free survival (NHR-FS) and extrahepatic recurrence free survival (EHR-FS). Clinical and imaging follow-up data was used as the reference standard.

**Results:**

Fifty-four patients with 90 lesions were selected. Univariable cox regression analysis resulted in eight models. Multivariable analysis revealed that after adjusting for lesion size and the approach of the procedure, none of the metabolic parameters were associated with LTP-FS or EHR-FS. Percutaneous approach was significantly associated with a shorter LTP-FS. It was demonstrated that lower values of SUL_peak_, SUL_max_, SUL_mean_ , and cSUL_mean_ are associated with a significant better NHR-FS, independent of the lesion size and number and prior chemotherapy.

**Conclusion:**

We found no association between the metabolic parameters on pre-ablation ^18^F-FDG PET/CT and the LTP-FS. However, low values of the metabolic parameters were significantly associated with improved NHR-FS. The clinical implication of these findings might be the identification of high-risk patients who might benefit most from adjuvant or combined treatment strategies.

**Electronic supplementary material:**

The online version of this article (doi:10.1007/s00259-017-3637-0) contains supplementary material, which is available to authorized users.

## Introduction

In recent years, many new treatment modalities have been established in the field of liver-directed oncologic interventions. Local tumour destruction by means of thermal ablation therapy has emerged as a safe and effective treatment modality for patients with colorectal liver metastases (CLM) who are not surgical candidates [1]. Radiofrequency ablation (RFA) and Microwave ablation (MWA) are considered safe ablation techniques with the ability to accomplish local disease control as shown in large cohort studies and meta-analyses [2, 3]. Furthermore, thermal ablation is frequently performed combined with concomitant liver resection [4].

Although ablation therapy has been proven to be effective in many cases, recurrent disease is a frequently reported problem that jeopardizes patients’ prognosis [1, 5]. Recurrent disease can be detected in the periphery of the ablation zone after successful ablation and is referred to as local tumour progression (LTP) [6]. The reported rates of LTP can be as high as 60% of lesions [1]. Recurrent disease also includes intra- and extrahepatic disease, occurring in 56% and 44% of patients, respectively [5, 6]. Identifying robust prognostic biomarkers might help to predict more accurately which eligible patients for thermal ablative therapy are at the highest risk for recurrent disease.

Several studies have investigated the role of ^18^F-fluorodeoxyglucose (^18^F-FDG) positron emission tomography/computed tomography (PET/CT) for selection and staging of patients who are considered for ablative treatment of liver lesions [7]. The role of ^18^F-FDG PET/CT has only been investigated in terms of detection of hepatic and extrahepatic lesions for staging of patients or for treatment evaluation. However, studies have shown that ^18^F-FDG PET specific parameters may have a prognostic role in assessing survival of patient with primary or secondary liver malignancies undergoing curative treatment [8, 9]. This association has also been investigated for other malignancies and demonstrated increased tumour aggressiveness with higher ^18^F-FDG uptake in tumour lesions [10–12].

Up to now, the role of metabolic parameters as imaging biomarkers for recurrent disease following ablative therapy has not been investigated. However, imaging biomarkers can offer a risk stratification tool to identify high-risk patients and allow individualized treatment strategies in order to improve patients’ survival. The aim of this retrospective cohort study was to evaluate the role of metabolic parameters in predicting the prognosis in terms of LTP, intrahepatic, and extrahepatic recurrent disease in patients with CLM treated by means of curative-intent thermal ablation therapy. We hypothesized that an aggressive tumour lesion was associated with a higher metabolic activity, resulting in a higher chance of recurrent disease.

## Patients and methods

This retrospective study was reviewed by the institutional review board, and the requirement to obtain informed consent was waived. We retrospectively identified and included all consecutive patients with CLM treated with percutaneous or open RFA or MWA (either alone or combined with liver resection) in Antoni van Leeuwenhoek Hospital Amsterdam, between January 2008 and July 2015. A baseline ^18^F-FDG PET/CT scan performed within 2 months prior to ablation therapy was an inclusion criterion and all ablation procedures were performed in a curative setting. We excluded tumour lesions that were not assessable on the baseline ^18^F-FDG PET/CT based on visual inspection. The decision for treatment was made during multidisciplinary tumour-board meeting consisting of at least one experienced liver surgeon, an intervention radiologist, a medical oncologist and a nuclear physician. The main indications for thermal ablation therapy were surgical resection (alone) technically not possible due to multilobar disease or previous liver resection; patient comorbidities could not ensure a safe surgical procedure; and patient preference for minimally invasive treatment. For each procedure, data on clinical and procedural characteristics were collected. Given prior reports of lower ^18^F-FDG uptake in mucinous type colorectal carcinoma [13], data on histopathology subtypes (mucinous versus non-mucinous type) of the primary colorectal carcinoma was collected as well.

### Thermal ablation therapy

Percutaneous thermal ablation procedure was performed under epidural anesthesia using CT-guidance for needle positioning and evaluation of the ablation zone. The open procedure was performed under general anesthesia, using ultrasound guidance for needle positioning. Until 2010, for RFA, the systems used were Covidien Cool-Tip RF Ablation system with switching controller. Thereafter, a Cool-Tip RF Ablation E-series system (Covidien, Mansfield MA, USA) was used. From August 2012, thermal ablation was also performed by means of MWA using an Emprint ablation system (Covidien, Mansfield, MA, USA). In case of RFA, either a single or cluster antenna was used depending on the required ablation volume. Thermal ablation procedure was performed by two experienced interventional radiologists and according to international guidelines [6] ensuring ≥ 5 mm rim of coagulated healthy liver tissue around the tumour lesion.

### Follow-up

The standard imaging follow-up schedule consisted of a tri-phasic CT scan within one month in case of percutaneous approach or a ^18^F-FDG PET/CT within 6–8 weeks in case of open ablation procedure. Subsequently, imaging evaluation by means of MRI, CT, or ^18^F-FDG PET/CT took place every 3 months until 1-year follow-up and thereafter biannually. All patients were followed at least 12 months after the ablation procedure for detection of recurrent disease. After that, patients were followed until the last follow-up visit or death. The follow-up time was defined as the time between the date of the ablation procedure and the development of recurrent disease or the last date on which follow-up was performed. Overall survival (OS) was defined as interval from ablation to death.

### ^18^F-FDG PET/CT and quantitative analysis

Whole-body ^18^F-FDG PET was performed in one institute on two Gemini TOF scanner (Philips Healthcare, Best, the Netherlands) at 2 min per bed position in 3-dimensional mode. Both scanners were EARL compliant. A graph presenting the recovery curves is included in the supplemental material (Supplemental material, Fig. S1). A low-dose CT was used for attenuation correction and anatomical correlation. Patients were prepared in concordance with the EANM guidelines [14] for tumour imaging. ^18^F-FDG was injected intravenously using a body mass index (BMI) based dosage scheme. The dose varied between 190 and 240 MBq depending on a BMI higher or lower than 28. In patients with very high or low BMI, the administered dose was in consultation with the physician. Scanning commenced 60 min after administration (mean 66 +/− 14 min).

The quantitative analysis was performed in concordance with the PET Response Criteria in Solid Tumours (PERCIST 1.0) [15] Calculation of the standardized uptake values corrected for lean body mass (SUL) was performed using ROVER evaluation software (ABX GmbH, Radeberg, Germany). The SUL_peak_, SUL_mean_, partial volume corrected SUL_mean_ (cSUL_mean_), SUL_max_ , and the total lesion glycolysis (TLG) were collected for all target lesions. The method for partial volume correction is previously described by Hofheinz et al. [16].

Delineation of liver lesions can be challenging due to the relatively high physiological accumulation in healthy liver tissue. Therefore, the mean SUL of background activity (SUL_bckgr_) was measured by placing a 3-cm diameter spherical volume of interest (VOI) in the healthy liver tissue; the tumour SUL_peak_ was determined in a 1-cm^3^ spherical VOI_peak_. According to the PERCIST 1.0 criteria, a baseline tumour SUL_peak_ has to be ≥1.5 times the SUL_bckgr_ of liver tissue plus two times its standard deviation (SD), otherwise quantification can be unreliable. Still, due to the limited number of patients, all measurements were included in the published analysis, also if the tumour SUL_peak_ was below this threshold. A segregated data analysis was performed for measurements that adhered to the strict PERCIST 1.0 criteria (Supplemental material, Table S1).

The threshold for the metabolic tumour volume delineation was set at 70% of the tumour SUL_peak_ (VOI_70_). However, this approach sometimes resulted in visually inaccurate delineations, especially when VOI_70_ was much smaller than the VOI_1.5bckgr+2SD_. In these cases, metabolic tumour volume was redefined as SUL_bckgr_ plus two times SD (*n* = 4 cases). The mean tumour SUL (SUL_mean_) is a direct derivative of this metabolic tumour volume. For the TLG, VOI threshold was defined as SUL_bckgr_ plus two times SD (VOI_bckgr+2SD_) as recommended by the PERCIST 1.0. The latter threshold was not used for the entire dataset since this threshold frequently resulted in inclusion of a large part of the healthy liver tissue in the VOI, especially when SUL_peak_ was much larger than the VOI_1.5bckgr+2SD_. Visual inspection was performed by comparing the VOI with the lesion margins as detected on a recently performed contrast enhanced CT. Partial volume correction was implemented for each measurement to account for underestimation of the SUL values. For patient-based analysis, the most metabolically active tumour lesion was used as target lesion.

### Definitions of outcome

Recurrent disease was stratified in site of recurrence: LTP, intrahepatic, and extrahepatic recurrence. LTP was the primary endpoint of the study and defined as appearance of tumour foci at the edge of the ablation zone (up to 1 cm from the edge), after at least one contrast-enhanced follow-up study has documented adequate ablation and an absence of viable tissue in the target tumour surrounding ablation margin [6]. Accordingly, lesions in which residual unablated tumour was detected on initial follow-up imaging, were excluded from analysis. Secondary endpoints were new hepatic recurrence (NHR) and extrahepatic recurrence (EHR). NHR was defined as new tumour foci outside the ablation zone (>1 cm distance from the ablation zone) in other parts of the liver and EHR was defined as new metastases in other organs than the liver (excluding patients in whom extrahepatic disease was present at baseline).

### Reference standard

Follow-up imaging (MRI, CT, or ^18^F-FDG PET/CT) and clinical data were used as the reference standard. The reports of all post-ablation imaging and the scans were evaluated prospectively by the local investigator in order to evaluate the presence of LTP, NHR, or EHR. In case of ambiguity between the imaging reports and the images, the scans were discussed with an experienced intervention radiologist (W.P.) in order to reach consensus. The evaluation of follow-up imaging was blinded for data of the baseline ^18^F-FDG PET/CT scan.

### Statistics

Descriptive analysis was performed to summarize patient characteristics and treatment characteristics, as well as the metabolic parameters. The median LTP-free survival (LTP-FS), NHR-free survival (NHR-FS), and the EHR-free survival (EHR-FS) were calculated using Kaplan-Meier method. For LTP-FS, analysis was performed based on a per-lesion basis and the NHR-FS and EHR-FS analyses were performed on a per-patient basis where the target lesion was defined as the lesion with the highest SUL_peak_ at baseline.

Univariable cox regression analysis was performed for each metabolic parameter as a risk factor associated with LTP-FS, NHR-FS, or EHR-FS. The metabolic parameter of interest was entered in a multivariable model if the P-value was ≤0.25. Multivariable cox regression analysis was undertaken to evaluate the prognostic potential of the metabolic parameter of interest for prediction of LTP-FS, adjusted for potential effect modifiers such as tumour size, approach of the procedure (open vs. percutaneous), and type of ablation therapy (RFA vs. MWA). Similar analysis was performed for NHR-FS and EHR-FS, adjusted for tumour number and size. The inclusion of covariates in the model was based on clinical relevance. The event per variable rule was used to decide on the appropriate number of variables in the model [17]. In order to investigate the effect of prior chemotherapy on ^18^F-FDG uptake of tumour lesions, a subset analysis was performed in the chemo-naïve patients. Chemo-naïve was defined as no chemotherapy at least one year prior to ablation therapy.

The proportional hazard assumption for the cox regression model was tested by means of a goodness-of-fit test using chi-square statistics computed for each variable in the model, and adjusted for the other variables in the model. To address the problem of multicollinearity, a correlation coefficient matrix was calculated, with values >0.8 suggesting collinearity between independent variables. In order to obtain clinical relevant results, the median value of the metabolic parameters in the cohort was initially used to retrieve a cut-off value for each parameter. In case of LTP-FS, the median values were based on a per-lesion analysis and in case of NHR-FS and EHR-FS, the median values were based on a per-patient analysis. The multivariable cox regression analysis was performed again including the dichotomized variables. Differences in the survival of the two groups (low and high metabolic value), adjusted for covariates in the model was demonstrated using Kaplan-Meier curves along with the 1-year survival rates. Statistical analyses were performed using RStudio version 3.1.2 open-source software. A P-value <0.05 was considered as statistically significant.

## Results

### Patient and tumour characteristics

**Fig. 1:**
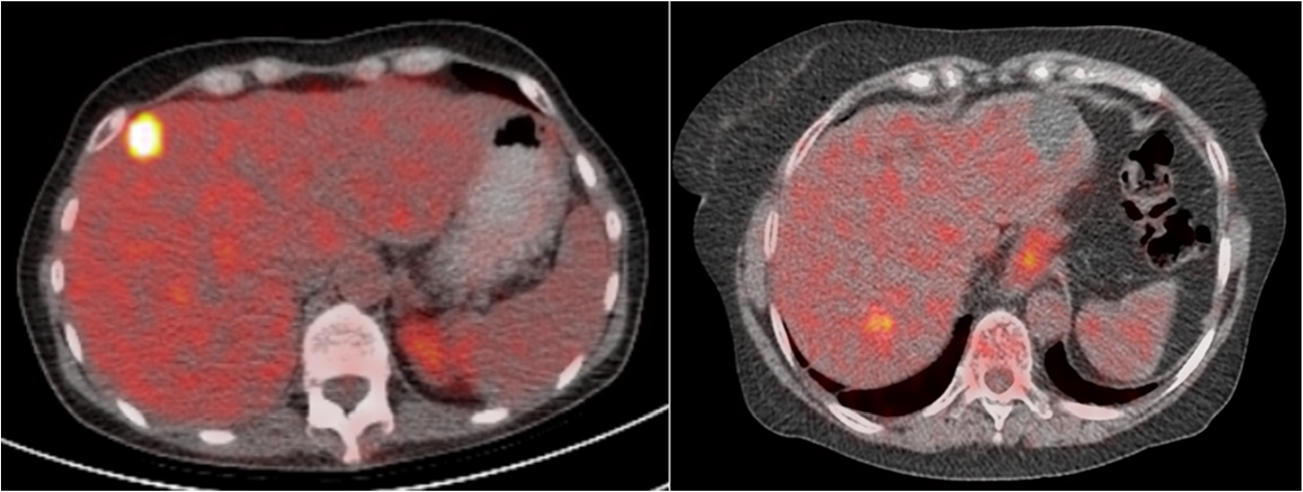
PET/CT imaging of a patient with high (left panel) versus low (*right panel*) ^18^F-FDG uptake in the tumour lesion with the median SUL_peak_ value as cut-off point (median SUL_peak_ of 4.8)

A total of 54 patients underwent 60 thermal ablation procedures and met the inclusion criteria for the study. During the 60 ablation procedures, 90 lesions were ablated. Table 1 summarizes the demographics and the tumour characteristics. The patient characteristics are based on the number of patients, the tumour characteristics are based on the lesion numbers and the procedure characteristics based on the number of procedures. Data on histopathology subtypes of the primary colorectal carcinoma showed only one patient with the mucinous type. RFA was used for ablation of 72 lesion (80%) and MWA for ablation of 18 (20%) lesion. Of the 60 ablation procedures, 31 (52%) were performed open and 29 (48%) percutaneously. The number of lesion on the preoperative imaging ranges between one and four lesions and of these, 17 lesions were resected during a combined procedure (Table 1). The median lesion size was 18 mm (7–55 mm) and 14 (23%) lesions were located ≤1 cm from large vessels. In three patients, incomplete ablation/residual disease was detected on the first follow-up scan in four lesions, so these lesions were excluded from the analysis for LTP-FS. Although all patients had liver dominant disease, extrahepatic disease was present at baseline in 11 patients (20%). These patients were excluded from the analysis for the EHR-FS. According to the PERCIST 1.0 criteria, 68 out of 90 lesions were defined as assessable target lesions (baseline tumour SUL_peak_ ≥1.5 times the SUL_bckgr_ of liver tissue plus two times its SD). This resulted in 47 patients for the NHR-FS analysis and 38 patients for the EHR-FS analysis according to PERCIST 1.0 (Supplemental material). Figure 1 demonstrates PET/CT imaging of a patient with high versus low ^18^F-FDG uptake in the tumour lesion.

**Table 1:** Demographics and tumour characteristics

Characteristic	N (%)
Number of patients	54
Number of procedures	60
Number of lesions	90
Age (median), year	62 (40–84, IQR 14)
Gender (Male/female)	33 (61)/21 (39)
RFA/MWA (lesion-based)	72 (80)/18 (20)
Open/Percutaneous approach (procedure-based)	31 (52)/29 (48)
ASA (n = 54)	
1	19 (35)
2	27 (50)
3	2 (4)
Missing	6 (11)
Comorbidity (n = 54)	
Hearth disease	5 (9)
Pulmonary disease	4 (7)
Renal disease	1 (2)
Other	9 (17)
Extrahepatic disease (n = 54)	11 (20)
Pulmonary	8 (15)
Lymph node	2 (4)
Peritoneal	1 (2)
Prior chemotherapy	28 (52)
Within 1 year from treatment	8 (15)
Adjuvant chemotherapy	
Within 6 months after treatment	11 (20)
Directly after treatment	2 (4)
Number of lesions on imaging (n = 60)^a^	
1	33 (55)
2	13 (22)
3	8 (13)
4	6 (10)
Number of lesions ablated (n = 60)	
1	41 (68)
2	10 (17)
3	7 (12)
4	2 (3)
Lesion size in mm (median, n = 90)	18 mm, range 7–55 mm, IQR 11 mm
Distance to large vessel ≤1 cm (n = 90)	14 (23)
Metabolic parameter (based on all lesions)	
SUL_peak_	4.8 (1.7–10.2, IQR 2)
SUL_mean_	4.0 (1.6–9.1, IQR 1.6)
cSUL_mean_	6.6 (2.3–19.8, IQR 3.7)
SUL_max_	5.4 (1.9–12.6, IQR 2.3)
TLG	23.7 (2.8–305.2, IQR 31.9)
SUL_mean_ of normal liver	2.0 (1.3–2.4, IQR 0.3)

^a^ Of these, 17 out of 107 lesions were resected during an combined procedure. ASA American Society of Anesthesiologists; IQR interquartile range; MWA microwave ablation; RFA radiofrequency ablation; TLG total lesion glycolysis

### Survival characteristics

The median follow-up time was 29.3 months (range 5.8–91.8 months). Lesion-based analyses resulted in a LTP rate of 46.5% (40 out of 86 lesions). Of these, 36 out of 86 (41.9%) were identified within 1 year and 20 out of 86 (23.3%) were identified within 6 months following the ablation procedure. The 6-month and 1-year LTP-FS were 80.2% (72.2–89.1) and 58.1% (48.3–69.7), respectively. The median number of follow-up moments within 1-year was 4.5 (range 3–8) in patients that did not develop LTP and 4 (range 2–8) in patients with LTP within 1 year. Thirty-two patients developed NHR (59.0%) and 24 out of 43 patients (56.0%), developed EHR during the course of the disease. The 1-year and 3-year NHR-FS and EHR-FS were 47.4% (35.7–63.0), 37.1% (25.4–54.0) and 62.6% (49.7–79.0), 41.9% (29.1–60.4), respectively. Twenty-one patients (39%) died during the entire follow-up. The median OS was 49.3 months with a 1-year and 5-year survival rate of 94.1% (87.9–100.0) and 28.0% (13.6–57.6), respectively.

### Cox regression analysis

The univariable analysis resulted in three models for LTP-FS analysis, four models for NHR-FS and one model for EHR-FS (Tables 2 and 3). The type of ablation treatment (RFA vs. MWA) did not meet the proportional hazard assumption and was not found to be an effect-modifier for the relation between the metabolic activity and LTP-FS. Therefore, this variable was not included in the multivariable model. The chi-square test resulted in a P-value >0.05 for the remaining categorical variables in the models. The correlation coefficient between the metabolic parameters of the hepatic metastases was only weakly correlated with lesion size (r <0.40) suggesting no complications caused by multicollinearity in the models.

**Table 2:** Univariable cox regression analysis for metabolic parameters as risk factors associated with LTP-FS, NHR-FS and EHR-FS

	LTP-FS HR (95% CI) *P*-value		NHR-FS HR (95% CI) *P*-value		EHR-FS HR (95% CI) *P*-value	
Metabolic parameter	Not assessable for PERCIST	Assessable for PERCIST	Not assessable for PERCIST	Assessable for PERCIST	Not assessable for PERCIST	Assessable for PERCIST
SUL-peak	0.92 (0.76–1.38) *0.38*	0.87 (0.68–1.11) 0.25*	1.31 (1.04–1.66) 0.02*	1.40 (1.07–1.82) 0.02*	1.17 (0.89–1.55) *0.26*	1.22 (0.88–1.69) 0.23*
SUL–max	0.92 (0.78–1.08) *0.30*	0.86 (0.69–1.08) 0.19*	1.21 (0.99–1.48) 0.06*	1.26 (1.00–1.59) 0.05*	1.13 (0.89–1.43) *0.34*	1.15 (0.87–1.54) *0.33*
SUL–mean	0.91 (0.72–1.14) *0.41*	0.85 (0.63–1.14) *0.28*	1.45 (1.08–1.95) 0.01*	1.60 (1.15–2.23) 0.01*	1.14 (0.81–1.61) *0.44*	1.19 (0.79–1.78) *0.41*
cSUL–mean	0.95 (0.85–1.05) *0.31*	0.92 (0.81–1.05) 0.23*	1.18 (1.05–1.33) 0.01*	1.23 (1.08–1.40) 0.01*	1.05 (0.90–1.22) *0.53*	1.06 (0.90–1.26) *0.47*
TLG	1.00 (0.99–1.01) *0.94*	1.00 (0.99–1.01) *0.94*	1.00 (1.00–1.01) *0.88*	1.00 (1.00–1.01) *0.78*	1.00 (0.99–1.01) *0.57*	1.00 (0.99–1.01) *0.57*

EHR-FS extrahepatic recurrence free survival; LTP-FS local tumour progression free survival; NHR-FS new hepatic recurrence free survival. *Relevant metabolic parameters used in the multivariable models are marked

**Table 3:** Multivariable cox regression models for LTP-FS (models 1–3), NHR-FS (models 4–7) and EHR-FS (model 8)

Model	Outcome variable	Covariates	Hazard ratio (95% CI)	*P*	Hazard ratio (95% CI)	*P*
			All patients		Chemo-naive patients	
Model 1	LTP-FS	Lesion size Percutaneous approach SUL-peak	1.03 (0.99–1.07) 2.34 (1.25–4.38) 0.88 (0.71–1.08)	*0.104* *0.008* *0.214*	*NA*	
Model 2	LTP-FS	Lesion size Percutaneous approach SUL -max	1.03 (0.99–1.07) 2.33 (1.24–4.37) 0.89 (0.74–1.06)	*0.108* *0.008* *0.186*	*NA*	
Model 3	LTP-FS	Lesion size Percutaneous approach cSUL-mean	1.03 (0.99–1.06) 2.32 (1.23–4.35) 0.93 (0.83–1.05)	*0.131* *0.009* *0.246*	*NA*	
Model 4	NHR-FS	Lesion size Lesion number SUL -peak	0.97 (0.94–1.01) 1.15 (0.84–1.58) 1.38 (1.09–1.74)	*0.168* *0.376* *0.007*	0.97 (0.94–1.02) 1.25 (0.92–1.71) 1.33 (1.00–1.78)	*0.273* *0.156* *0.048*
Model 5	NHR-FS	Lesion size Lesion number SUL -max	0.98 (0.94–1.02) 1.15 (0.84–1.58) 1.25 (1.02–1.53)	*0.252* *0.368* *0.029*	0.98 (0.95–1.02) 1.26 (0.92–1.72) 1.20 (0.95–1.53)	*0.382* *0.145* *0.123*
Model 6	NHR-FS	Lesion size Lesion number SUL -mean	0.97 (0.93–1.01) 1.12 (0.82–1.54) 1.60 (1.18–2.17)	*0.106* *0.477* *0.003*	0.97 (0.93–1.01) 1.22 (0.89–1.66) 1.59 (1.06–2.40)	*0.177* *0.222* *0.025*
Model 7	NHR-FS	Lesion size Lesion number cSUL-mean	0.98 (0.94–1.02) 1.08 (0.79–1.48) 1.20 (1.06–1.35)	*0.214* *0.624* *0.003*	0.98 (0.94–1.02) 1.18 (0.86–1.61) 1.20 (1.02–1.40)	*0.315* *0.311* *0.027*
Model 8	EHR-FS	Lesion size SUL-peak	0.98 (0.94–1.03) 1.22 (0.92–1.62)	*0.400* *0.174*	*NA*	

CI confidence interval; CN Chemo-naïve patients; EHR-FS extrahepatic recurrence free survival; LTP-FS local tumour progression free survival; NA not applicable; NHR-FS new hepatic recurrence free survival

Multivariable analysis revealed that after adjusting for lesion size and the approach of the procedure, none of the metabolic parameters were associated with LTP-FS (Table 3). Percutaneous approach was significantly associated with a shorter LTP-FS (hazard ratio of 2.3, *P* <0.01), independent of the lesion size. Models 4–7 demonstrated that low values of SUL_peak_, SUL_max_, SUL_mean_ , and cSUL_mean_ are significantly associated with a better NHR-FS, independent of the lesion size and number (Table 3). In other words, new hepatic lesions were earlier observed in patients with more metabolically active tumours. Of these, the SUL_mean_ had the highest hazard ratio for a shorter NHR-FS (model 6, hazard ratio of 1.60, *P* = 0.003). Based on the hazard ratio and the significance level, the cSUL_mean_ did not seem to be superior compared to the SUL_mean_ (Table 3). The results of the multivariable analysis did not reveal any association between SUL_peak_ , adjusted for lesion size, and the EHR-FS. The multivariable analysis was performed separately in data according to PERCIST 1.0 and results were in line with the results of the analysis in the entire dataset (Supplemental material, Table S1).

**Fig. 2:**
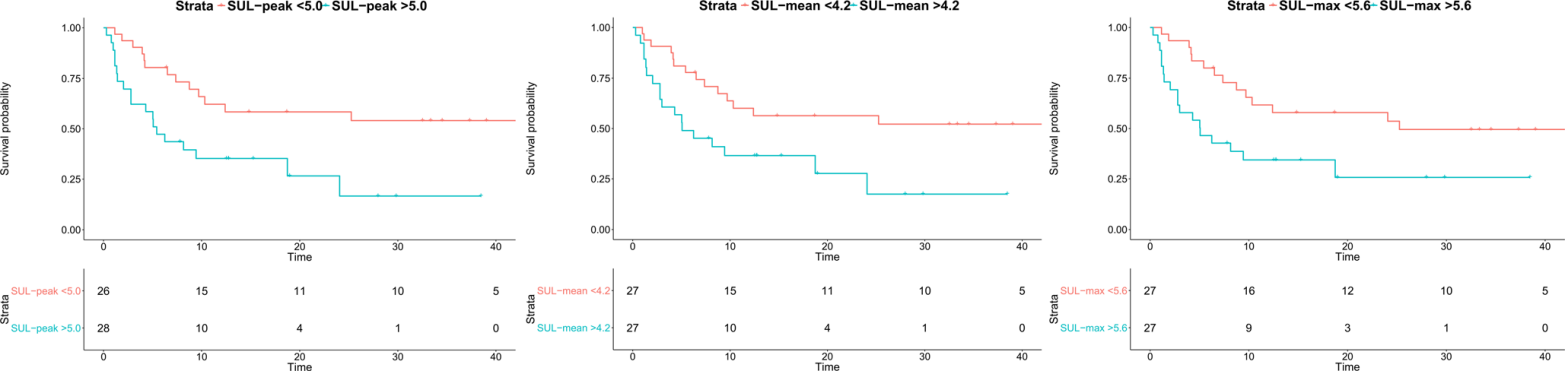
Adjusted Kaplan-Meier curves for NHR-FS with dichotomized values of the metabolic parameters SUL_peak_, SUL_mean_ , and SUL_max_. The 1-year NHR-free rate was 62.2% (95% CI 46.1–83.9) and 35.2% (95% CI 20.8–59.9) for SUL_peak_ lower and higher than 5.0 respectively, 60.1% (95% CI 44.1–81.9) and 36.6% (95% CI 21.6–61.9) for SUL_mean_ lower and higher than 4.2 and 61.2% (95% CI 45.3–82.9) and 61.7% (95% CI 45.7–83.2) and 34.4% (95% CI 20.0–59.2) for SUL_max_ lower and higher than 5.6, respectively

**Fig. 3:**
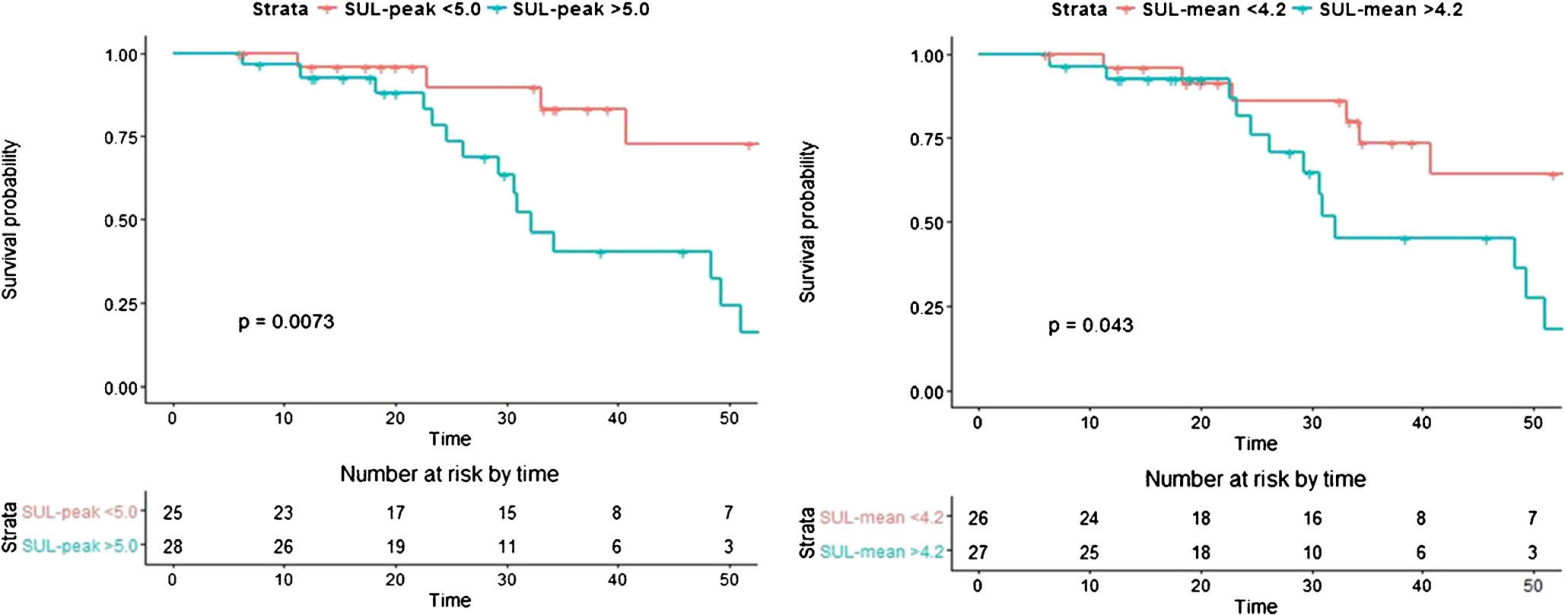
Kaplan-Meier curves for OS with dichotomized values of the metabolic parameters SUL_peak_ (*left*) and SUL_mean_ (*right*)

The chosen cut-off values for SUL_peak_, SUL_max_, SUL_mean_ , and cSUL_mean_ were 5.0, 5.6, 4.2, and 6.8, respectively. Multivariable cox regression analysis demonstrated a hazard ratio of respectively 2.7, 2.5, 2.4, and 2.1 for NHR-FS, independent of lesion size and number (Table 4). This can be interpreted as a 2.7 times higher risk of developing intrahepatic disease per unit time in patients with SUL_peak_ >5.0. Only the cut-off value for cSUL_mean_ did not remain significant after dichotomizing the metabolic parameter (*P* = 0.053). Kaplan-Meier survival curves were plotted for NHR-FS stratified for high and low SUL_peak_, SUL_max_ , and SUL_mean_, adjusted for tumour number and size (Fig. 2). The mean 1-year NHR-free rates for the high versus low values of the metabolic parameters was 35% versus 61%, respectively. Additionally, Kaplan-Meier analysis was performed in order to evaluate differences in OS stratified for high versus low value of the SUL_peak_ and the SUL_mean_. Figure 3 illustrates the significant differences in OS of patients with a preoperative high versus low value of the SUL_peak_ and the SUL_mean_. The calculated 3-year survival rates were 83.3% versus 40.3% in the low versus high SUL_peak_ group. Accordingly, the 3-year survival rate in the low versus high SUL_mean_ group was 73.5% versus 45.4% respectively.

**Table 4:** Multivariable cox regression models for NHR-FS with dichotomized values of the metabolic parameters

Model	Covariates	Hazard ratio (95% CI)	*P*	Hazard ratio (95% CI)	*P*
		All patients		Chemo-naive patients	
Model 4	Lesion size Lesion number SUL-peak >5.0	0.97 (0.93–1.01) 1.17 (0.85–1.61) 2.66 (1.22–5.77)	*0.104* *0.330* *0.014*	*0.98 (0.93–1.02)* *1.29 (0.95–1.77)* *1.96 (0.84–4.60)*	*0.251* *0.107* *0.118*
Model 5	Lesion size Lesion number SUL-mean >4.2	0.97 (0.93–1.01) 1.17 (0.85–1.60) 2.38 (1.10–5.13)	*0.125* *0.336* *0.028*	*0.98 (0.94–1.02)* *1.28 (0.78–0.93)* *1.94 (0.83–4.50)*	*0.268* *0.129* *0.124*
Model 6	Lesion size Lesion number SUL-max >5.6	0.97 (0.93–1.01) 1.16 (0.84–1.59) 2.45 (1.15–5.20)	*0.159* *0.362* *0.020*	*0.98 (0.94–1.02)* *1.27 (0.79–0.93)* *1.51 (0.68–3.37)*	*0.428* *0.136* *0.316*
Model 7	Lesion size Lesion number cSUL-mean >6.8	0.98 (0.94–1.02) 1.03 (0.75–1.43) 2.13 (0.99–4.57)	*0.241* *0.841* *0.053*	*0.99 (0.95–1.03)* *1.22 (0.87–1.71)* *1.30 (0.57–2.97)*	*0.534* *0.240* *0.538*

### Analysis in chemo-naïve patients

Eight patients (15%) had received systemic therapy at least 1 year prior to ablation therapy. The univariable and multivariable analysis was repeated in the chemo-naïve patients (Supplemental material, Table S2). The univariable analysis in the chemo-naïve patients resulted in four models corresponding with models 4, 5, 6, and 7 (Table 3). The results of the multivariable analysis in the chemo-naïve patients showed that low values of SUL_peak_, SUL_mean_ , and cSUL_mean_, but not SUL_max_, were significantly associated with a better NHR-FS. However, the dichotomized values of the SUL_peak_, SUL_mean_ , and cSUL_mean_ did not remain significant in the multivariable analysis (Table 4).

## Discussion

This study showed that the metabolic parameters calculated based on the pre-ablation ^18^F-FDG PET/CT were not associated with LTP-FS. In the multivariable model, the percutaneous approach of the procedure remained the most significant predictor of LTP. An important finding was that the metabolic parameters were independent prognostic risk factors for NHR-FS, even in chemo-naïve patients. Remarkably, none of the metabolic parameters were associated with EHR-FS.

Our results suggest that the aggressiveness of the tumour lesions as expressed by means of the metabolic activity is not associated with shorter LTP-FS after thermal ablation therapy. A possible explanation might be that appearance of LTP highly depends on technical aspects of the procedure and tumour size, rather than the metabolic activity. Evidently, the success of ablation therapy greatly depends on the correct positioning of the ablation probe and visibility of the tumour lesion [18]. During an open procedure, localization of the lesion is done by means of intraoperative ultrasound. Use of ultrasound imaging eliminates attenuation by the skin and subcutaneous tissue and ensures a wider window, resulting in an improved visibility and image resolution. Another benefit of the open procedure is the improved controlled positioning of the probe allowing insertion of the probe at different angles with mobilization of the liver if necessary [18].

Despite the differences in the LTP rate, percutaneous ablation is considered the least invasive method and is recommended over the open approach due to the increased mortality and morbidity of the latter [3]. Obviously, this does not count for an ablation procedure that is combined with surgical liver resection [3] Moreover, it would be of interest whether and how the OS or the progression free survival is affected by the approach of the procedure. Unfortunately, our study design did not allow for an analysis for this purpose.

The reported LTP rates after ablation therapy vary widely between studies and comparison between cohorts is hampered due to analysis on a per-lesion or a per-patient basis. Reported lesion-based LTP rate is up to 43% [3] for open ablation and up to 52% for the percutaneous approach [3, 19]. Although higher than expected, the LTP rate in our cohort lies within the range of previous reported rates. Besides the approach of the procedure, the diameter of the lesion (>3 cm) and minimal ablation margin size are important known risk factors [19, 20]. In our cohort, lesion size was not considered as an exclusion criterion. As a result, lesions measuring >3 cm were also included which might explain our LTP rate. Furthermore, current evidence shows that an ablation margin between 5 and 10 mm is a prognostic factor for shorter LTP-FS and ideally, an ablation margin ≥10 mm should be endeavored [19]. Unfortunately, an ablation margin of ≥5 mm was aimed in our clinic.

The prognostic value of the metabolic parameters for time to progression of intrahepatic recurrence is an important finding since patients’ prognosis after ablation therapy seems to be more depending on intrahepatic recurrence rather than LTP. It was shown that LTP alone does not significantly affect the OS of patients treated by means of ablation therapy, but the pattern of disease recurrence does [21]. The OS of patients with LTP combined with intrahepatic recurrence was found to be worse compared to patients with LTP alone. A possible explanation is that a repeated ablation therapy, hence curative treatment, is still feasible in majority of patients with timely detected LTP.

Previous studies reported the prognostic significance of metabolic parameters for the survival of patients with surgically treated CLM [8, 22–24]. However, to our knowledge, this is the first study that investigated this for patients with CLM treated by means of curative-intent thermal ablation with stratified results for site of recurrence. We showed that a higher metabolic value is significantly associated with a worse NHR-FS. In case of a the SUL_peak_ >5.0, multivariable analysis showed a 2.7 times higher risk of developing intrahepatic disease per unit time. Since all PET imaging was performed using an EARL compliant PET scanner, the resulted cut-off values for the metabolic parameters can be useful in other EARL accredited centers [25].

Although we included only patients treated with thermal ablation therapy, our results are consistent with findings by other authors [8, 22–24]. A recent meta-analysis pooled the data on the prognostic significance of metabolic parameters and patients’ survival and found a significant association between a high pretreatment SUV and a poor OS (pooled hazard ratio of 1.24, 95% CI 1.06–1.45) [26]. Lee et al. investigated the prognostic significance of pre-treatment metabolic values and found a significant association between the SUV_peak_ of hepatic metastases and the recurrence free survival [8]. Other researchers conducted similar studies, but discrepancy in the reported results existed. According to Riedl et al. [22], the SUV_max_ measured on the pre-operative ^18^F-FDG PET was associated with a significant shorter OS, while Muralidharan et al. [24] did not find any association between the SUV_max_ or the SUV_mean_ and OS or recurrence free survival. The differences in the prognostic ability of the metabolic parameters reported by various authors might be the result of variability of technical and biological factors as well as heterogeneity of the population in the studies [27].

Nevertheless, the findings from previous studies, as well as present study show that higher metabolic activity of the tumour lesion is associated with a relatively poor outcome. PET imaging visualizes the increased glucose use by tumour cells using ^18^F-FDG as tracer. However, the cellular and molecular mechanisms that determine ^18^F-FDG uptake are poorly understood. Traditionally, histopathological characteristics are considered as reliable markers for biological aggressiveness [28]. The latter is considered a major determinant of clinical outcome of patients [28].

Careful selection of candidates for thermal ablation therapy is important and recommended by the international expert panel [3]. To maintain good survival rates, similar as after surgical resection of liver metastases, adjuvant systemic therapy should be acknowledged after curative thermal ablation [3]. However, it is not clear which patients benefit most from adjuvant systemic treatment. Previous studies showed the beneficial effect of adjuvant chemotherapy on the progression free survival [29–31]. The results of a randomized trial by Ruers et al. showed a significant improvement in progression free survival in patients treated with ablation therapy combined with systemic therapy compared to systemic therapy alone [29]. In the current study, a large difference in the 1-year NHR-free rate of patients with a high versus low metabolic value (35% versus 61%) was demonstrated. These findings might indicate that especially patients with highly metabolic active tumours, as measured based on the pre-procedural ^18^FDG-PET/CT, might benefit most from adjuvant systemic treatment or combined therapies.

In this study, we found no association between the metabolic activity and the EHD-FS. To our knowledge, no other studies have investigated the correlation of metabolic activity and extrahepatic disease recurrence. Other studies that investigated possible predictors of extrahepatic recurrence after curative-intent surgery, found the following characteristics associated with an increased risk: primary rectal tumour site, primary tumour lymph node metastasis, hepatic tumour size >5 cm, hepatic tumour number >4, as well as receipt of chemotherapy [32–35]. The lack of association between metabolic activity and extrahepatic disease in our cohort is remarkable as our results demonstrated a significant association between metabolic activity and OS. A possible explanation for our findings is the small sample size that was used for this analysis. Hence, the association between the metabolic activity and extrahepatic recurrence after curative-intent surgery needs to be further addressed in future studies with larger cohorts.

There are limitations to this study. The retrospective nature introduces the risk of selection bias. However, we used clearly defined inclusion and exclusion criteria and included all consecutive patients to mitigate this problem. The threshold for the metabolic tumour volume delineation was set at 70% of the tumour SUL_peak_ unless this approach resulted in visually inaccurate delineation. This was the case in four patients for whom a different threshold for delineation (SUL_bckgr+2SD_) was used. The use of different thresholds might have introduced bias in the reported results. However, the segregated analysis for measurements according to the PERCIST 1.0 criteria showed similar results as in the entire dataset. Also, the VOI_70_ threshold for tumour volume delineation was not according to EANM guidelines [13]. The post injection time varied quite (66 +/− 14 min) between patients which may have affected the tumour to normal-liver uptake ratio and subsequently the resulted metabolic value. Finally, although the SUV corrected for body weight is more often used in the clinical practice, we used the SUL values instead, because the latter is more consistent from patient to patient [36]

There is cumulative evidence on the prognostic significance of pretreatment ^18^F-FDG PET/CT. The call for more individualized approach to cancer treatment challenges future studies to investigate prospectively whether risk stratification of patients by means of metabolic parameters will lead to change of management and significant improvement in patients’ survival. Moreover, it should be recognized that the benefit of PET imaging can be extended by using different tracers for different biological features such as hypoxia level, which in turn is associated with negative effect on prognosis [37–39]. Perhaps this can lead to promising risk stratification tools that combine different biological imaging markers based on an entirely non-invasive method.

## Conclusion

Our findings add to the growing knowledge of the value of ^18^F-FDG PET/CT in the staging and evaluation setting of patients with CLM. We found no association between the metabolic parameters on pre-ablation ^18^F-FDG PET/CT and the LTP-FS. However, low values of the metabolic parameters were significantly associated with improved NHR-FS. In the era of tailored approach to patient care, the clinical implication of these findings is the identification of patients who might benefit most from adjuvant or combined treatment strategies.

## Electronic supplementary material

Below is the link to the electronic supplementary material.

ESM 1 (DOC 36 kb)

ESM 2 (DOC 34 kb)

ESM 3 (DOC 44 kb)
