# Supplementary material for: 18F-FDG PET as novel imaging biomarker for disease progression after ablation therapy in colorectal liver metastases
Source: Eur J Nucl Med Mol Imaging. 2017 Feb 8;44(7):1165–75. doi: 10.1007/s00259-017-3637-0 (PMC5434127; doi:10.1007/s00259-017-3637-0)
Supplement: Supplementary file 1 — (DOC 36 kb) [file 259_2017_3637_MOESM1_ESM.doc]

**Supplemental material**

**Table S1. Multivariable cox regression models for LTP-FS (models 1-3), NHR-FS (models 4-7) and HER-FS (model 8) with data according to PERCIST 1.0**.

|  | **Outcome variable** | **Covariates** | **Hazard ratio (95% CI)** | ***P*** |
| --- | --- | --- | --- | --- |
| Model 1 | LTP-FS | Lesion size  Percutaneous approach  SUL-peak | 1.03 (0.99-1.07)  2.43 (1.19-4.94)  0.82 (0.63-1.07) | *0.188*  *0.014*  *0.141* |
| Model 2 | LTP-FS | Lesion size  Percutaneous approach  SUL -max | 1.03 (0.99-1.07)  2.43 (1.19-4.96)  0.83 (0.66-1.05) | *0.219*  *0.014*  *0.119* |
| Model 3 | LTP-FS | Lesion size  Percutaneous approach  cSUL-mean | 1.03 (0.99-1.07)  2.44 (1.20- 4.95)  0.80 (0.58-1.09) | *0.186*  *0.014*  *0.158* |
| Model 4 | NHR-FS | Lesion size  Lesion number  SUL -peak | 0.98 (0.94-1.02)  1.22 (0.87-1.70)  1.47 (1.12-1.92) | *0.298*  *0.246*  *0.005* |
| Model 5 | NHR-FS | Lesion size  Lesion number  SUL -max | 0.99 (0.95-1.02)  1.21 (0.87-1.69)  1.31 (1.04-1.65) | *0.427*  *0.253*  *0.023* |
| Model 6 | NHR-FS | Lesion size  Lesion number  SUL -mean | 0.97 (0.93-1.01)  1.18 (0.85-1.65)  1.74 (1.24-2.44) | *0.196*  *0.322*  *0.001* |
| Model 7 | NHR-FS | Lesion size  Lesion number  cSUL-mean | 0.98 (0.94-1.02)  1.12 (0.81-1.56)  1.23 (1.08-1.40) | *0.398*  *0.482*  *0.002* |
| Model 8 | EHR-FS | Lesion size  SUL-peak | 0.99 (0.94-1.04)  1.24 (0.89-1.73) | *0.666*  *0.200* |
