# Supplementary material for: 18F-FDG PET as novel imaging biomarker for disease progression after ablation therapy in colorectal liver metastases
Source: Eur J Nucl Med Mol Imaging. 2017 Feb 8;44(7):1165–75. doi: 10.1007/s00259-017-3637-0 (PMC5434127; doi:10.1007/s00259-017-3637-0)
Supplement: Supplementary file 2 — (DOC 34 kb) [file 259_2017_3637_MOESM2_ESM.doc]

**Table S2. Univariable cox regression analysis for metabolic parameters as risk factors associated with LTP-FS, NHR-FS and HER-FS in chemo-naive patients only.**

|  | LTP-FS  HR (95% CI)  *P*-value | NHR-FS  HR (95% CI)  *P*-value | EHR-FS  HR (95% CI)  *P*-value |
| --- | --- | --- | --- |
| SUL-peak | 0.95  (0.77-1.16)  *0.60* | 1.26  (0.96-1.66)  *0.09** | 1.02  (0.75-1.39)  *0.89* |
| SUL-max | 0.94  (0.79-1.12)  *0.51* | 1.17  (0.93-1.47)  *0.18** | 1.00  (0.77-1.31)  *0.97* |
| SUL-mean | 0.95  (0.74-1.22)  *0.67* | 1.42  (0.98-2.05)  *0.06** | 0.94  (0.64-1.36)  *0.73* |
| cSUL-mean | 0.97  (0.86-1.09)  *0.57* | 1.19  (1.02-1.39)  *0.03** | 0.96  (0.81-1.13)  *0.60* |
| TLG | 1.00  (0.99-1.01)  *0.84* | 1.00  (0.98-1.01)  *0.60* | 1.00  (0.99-1.01)  *0.43* |

*Relevant metabolic parameters used in the multivariable models are marked
