# Supplementary material for: 18F-FDG PET as novel imaging biomarker for disease progression after ablation therapy in colorectal liver metastases
Source: Eur J Nucl Med Mol Imaging. 2017 Feb 8;44(7):1165–75. doi: 10.1007/s00259-017-3637-0 (PMC5434127; doi:10.1007/s00259-017-3637-0)

**Figure S1. The recovery curve presenting volume of the spheres against the recovery coefficient with the grey lines representing the EANM recommended values.**


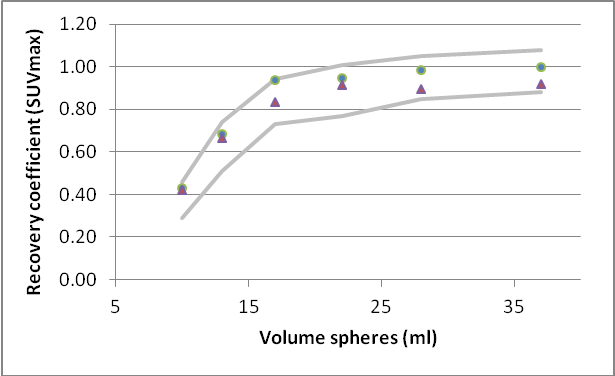

Supplement: Supplementary file 3 — (DOC 44 kb) [file 259_2017_3637_MOESM3_ESM.doc]
